# Normofractionated and moderately hypofractionated proton therapy: comparison of acute toxicity and early quality of life outcomes

**DOI:** 10.3389/fonc.2022.962697

**Published:** 2022-08-16

**Authors:** Maciej J. Pelak, Birgit Flechl, Eugen Hug, Razvan Galalae, Lisa Konrath, Joanna Góra, Piero Fossati, Carola Lütgendorf-Caucig, Slavisa Tubin, Rastko Konstantinovic, Ulrike Mock, Christoph Fussl, Petra Georg

**Affiliations:** ^1^ MedAustron Ion Therapy Center, Wiener Neustadt, Austria; ^2^ Medizinische Fakultät, Christian-Albrechts-Universität zu Kiel, Kiel, Germany; ^3^ Universitätsklinik für Radiotherapie und Radio-Onkologie, Landeskrankenhaus (LKH) Salzburg, Salzburg, Austria

**Keywords:** proton therapy, quality of life, toxicity, head and neck (H&N) cancer, re-irradiation (re-RT), hypofractionated radiotherapy

## Abstract

**Aim:**

Data on the safety of moderately hypofractionated proton beam therapy (PBT) are limited. The aim of this study is to compare the acute toxicity and early quality of life (QoL) outcomes of normofractionated (nPBT) and hypofractionated PBT (hPBT).

**Material and methods:**

We prospectively compared acute toxicity and QoL between patients treated with nPBT (dose per fraction 1.8–2.3 Gy, n = 90) and hPBT (dose per fraction 2.5–3.1 Gy, n = 49) in following locations: head and neck (H&N, n = 85), abdomen and pelvis (A&P, n = 43), and other soft tissue (ST, n = 11). The toxicities were grouped into categories—mucosal, skin, and other sites—and evaluated according to the Common Terminology Criteria for Adverse Events (CTCAE) version 4.03 at baseline, treatment completion, and 3 months after PBT completion. QoL was evaluated with the European Organisation for Research and Treatment of Cancer (EORTC) Quality of Life Questionnaire (QLQ)-C30 scale for all locations and additionally with EORTC QLQ-HN35 for H&N patients.

**Results:**

Overall, the highest toxicity grades of G0, G1, G2, and G3 were observed in 7 (5%), 40 (28.8%), 78 (56.1%), and 15 (10.8%) patients, respectively. According to organ and site, no statistically significant differences were detected in the majority of toxicity comparisons (66.7%). For A&P, hPBT showed a more favorable toxicity profile as compared to nPBT with a higher frequency of G0 and G1 and a lower frequency of G2 and G3 events (p = 0.04), more patients with improvement (95.7% *vs* 70%, p = 0.023), and full resolution of toxicities (87% vs 50%, p = 0.008). Skin toxicity was unanimously milder for hPBT compared to nPBT in A&P and ST locations (p = 0.018 and p = 0.025, respectively). No significant differences in QoL were observed in 97% of comparisons for QLQ-C30 scale except for loss of appetite in H&N patients (+33.3 for nPBT and 0 for hPBT, p = 0.02) and role functioning for A&P patients (0 for nPBT *vs* +16.7 hPBT, p = 0.003). For QLQ-HN35, 97.9% of comparisons did not reveal significant differences, with pain as the only scale varying between the groups (−8.33 *vs* −25, p = 0.016).

**Conclusion:**

Hypofractionated proton therapy offers non-inferior early safety and QoL as compared to normofractionated irradiation and warrants further clinical investigation.

## Introduction

Proton beam therapy (PBT) is an established radiation therapy modality that offers excellent tumor control or a favorable toxicity profile in selected indications. Fractionation schemes used in clinical practice are frequently similar and analogous to photon irradiation. Most commonly in use are ‘normofractioned’ doses of 1.8–2 Gy (relative biological effectiveness (RBE)) per fraction. In certain clinical situations, applying higher doses per fraction might be beneficial. These include re-irradiation of recurrent tumors, tumors of low assumed α/β ratio, and certain more aggressive neoplasms with expected increased resistance to radiation (1, 2). In these applications, photon radiation employs frequently moderate hypofractionation schemes (2.5–3.5 Gy per fraction); however, the reports on similar hypofractionation schemes by use of protons are limited, indicating possibly a certain hesitation of adaptation by proton therapy centers. One reason might be historical experiences resulting in significant acute toxicity increase by non-conventional accelerated radiotherapy schemes, in particular for head and neck tumors (3, 4). In addition, it is speculated that the RBE at the end of a range of protons (within the Bragg peak) is considerably higher than the RBE factor of 1.1 in general clinical use, thus contributing potentially to unexpected increased toxicity (5). Proton radiotherapy using both normo- and hypofractionated schemes is in routine clinical use at our institution. We decided to investigate the early safety of the latter by comparing acute toxicity outcomes and the quality of life (QoL) at corresponding time points between the two groups.

## Materials and methods

### Patient cohort and treatment specification

One hundred thirty-nine patients treated with curative intent and enrolled in the prospective registry study at our institution (REGI-MA-002015) (6) or participating in another prospective study (PRLI-MA-012016) were included (7). All patients had signed informed consent to participate in these studies, which were approved by the regional ethics board. For all patients, the Equivalent dose in 2-Gy fractions (EQD2) value (α/β = 10 assumed for all locations except for prostate for which α/β = 2 was considered) of prescribed total dose exceeded 54 Gy RBE. Detailed clinical patient and tumor characteristics are shown in **Table 1**. Of note is that our series included 50 patients (36%) receiving PBT as re-irradiation after having failed a prior course or radiotherapy. Treatment was delivered using pencil-beam-scanning protons, on average 5 days a week, once per day, for both normo- and hypofractionated schemes. Treatment was planned using RayStation (RaySearch Labs AG, Stockholm, Sweden) versions 6 to 8 (due to upgrades performed over time). The dose was computed with the Monte Carlo algorithm with a fixed RBE 1.1 ratio assumed without any additional modeling at the end of the Bragg peak range. The minimum number of beams used was three based on single-field optimization planning. Multi-field optimization technique was employed on an individual basis if it resulted in improved critical organ sparing without affecting plan robustness. **Table 2** displays the characteristics of proton therapy.

**Table 1:** Patient and tumor characteristics.

Parameter	No. of patients (%)					
	Head and neck(n = 85)		Abdomen and pelvis(n = 43)		Other soft tissue locations(n = 11)	
Sex						
Male	38 (44.7%)		27 (62.8%)	4 (63.6%)		
Female	47 (55.3%)		16 (37.2%)	7 (36.4%)		
Median age (range)	61.3 (13.7–87.6)		64.6 (25–90.7)	32 (5.2–66.8)		
Macroscopic tumor (GTV)						
No	27 (31.8%)		6 (14%)	6 (54.5%)		
Yes	58 (68.2%)		37 (86%)	5 (45.5%)		
Previous radiotherapy in the same area						
No	49 (57.6%)		31 (72.1%)	9 (81.8%)		
Yes	36 (42.4%)		12 (27.9%)	2 (18.2%)		
Tumor location						
	Sinonasal	33 (38.8%)	Prostate	20 (46.5%)	Paraspinal	5 (45.4%)
	Oral cavity	15 (17.6%)	Pelvic side wall and sacrum	14 (32.6%)	Thorax wall	3 (27.3%)
	Ear and mastoid	9 (10.6%)	Retroperitoneum and abdominal wall	6 (14.0%)	Breast	2 (18.2%)
	Parotid	9 (10.6%)	Other	3 (7.0%)	Extremity	1 (9.1%)
	Nasopharynx	7 (8.2%)				
	Oro- and hypopharynx	6 (7.1%)				
Histology						
	Squamous cell carcinoma	31 (36.5%)	Prostatic adenocarcinoma	20 (46.5%)	Sarcoma (soft tissue)	5 (45.4%)
	Adenoid cystic carcinoma	21 (24.7%)	Sarcoma (osteogenic)	8 (18.6%)	Desmoid tumor	3 (27.3%)
	Adenocarcinoma	11 (12.9%)	Sarcoma (soft tissue)	4 (9.3%)	Ewing Sarcoma	2 (18.2%)
	Sarcoma (soft tissue)	6 (7.1%)	Adenocarcinoma	5 (11.6%)	Chordoma	1 (9.1%)
	Sarcoma (osteogenic)	5 (5.9%)	SCC	4 (9.3%)		
	Other	11 (12.9%)	Other	2 (4.7%)		

GTV, gross tumor volume; SCC, squamous cell carcinoma.

**Table 2:** Characteristics of proton therapy.

Parameter		No. of patients	
	Head and neck(n = 85)	Abdomen and pelvis(n = 43)	Other soft tissue locations(n = 11)
PBT fractionation			
Normofractionated	63 (74.1%)	20 (46.5%)	7 (63.6%)
Hypofractionated	22 (25.9%)	23 (53.5%)	4 (36.4%)
Prescription concept			
Single CTV only	9 (10.6%)	8 (18.6%)	1 (9.1%)
SIB	34 (40%)	21 (48.9%)	4 (36.4%)
Sequential	42 (49.4%)	14 (32.5%)	6 (54.5%)
Median EQD2* in Gy RBE (range)	71.5 (58.5–80.8)	67.7 (60-78.3)	60.2 (55.3–80.8)
Median total dose in Gy RBE (range)			
Normofractionated	70 (60–76)	74.4 (60–79.2)	61.2 (60–70)
Hypofractionated	66 (54–77.1)	62 (60–69)	58.75 (51–60)
Median dose per fraction in Gy RBE (range)			
Normofractionated	2 (1.8–2.3)	2 (1.8–2.2)	2 (1.8–2.2)
Hypofractionated	3 (2.57–3.1)	3 (2.5–3.1)	3 (2.5–3)

SIB, simultaneous integrated boost; PBT, proton beam therapy; CTV, clinical target volume; RBE, relative biological effectiveness.

*α/β ratio of 10 used for calculation.

### Toxicities and quality of life assessment

Pre-existing symptoms were recorded at the baseline interview, and new toxicities were recorded at weekly control visits during PBT, at its completion, and during the 3-month follow-up control visit. All toxicities were scored according to the Common Terminology Criteria for Adverse Events (CTCAE) scale version 4.0. For the purpose of the statistical analysis, the toxicities were grouped into categories: mucosal, skin, and other (not associated with skin and mucosa). The highest grades observed since therapy started and 3-month follow-up were recorded. Additionally, it was assessed if toxicities observed during the treatment resolved or at least improved at the 3-month follow-up and if new toxicities appeared between completion of therapy and at 3-month follow-up (delayed acute adverse events).

Quality of life was evaluated using standardized EORTC QLQ-C30 questionnaires for all tumor groups and, in addition, the organ-specific EORTC QLQ-HN35 module for head and neck patients. EORTC QLQ-C30 is a validated instrument evaluating a patient’s global health, functioning (physical, role, emotional, cognitive, and social), most common disease symptoms (fatigue, nausea, pain, dyspnea, insomnia, loss of appetite, constipation, and diarrhea), and other factors. EORTC QLQ-HN35 is an organ-specific module for the assessment of disease symptoms attributable to head and neck tumors including pain, swallowing, sensual problems, speech, social contacts, eating, dental condition, and xerostomia. Scores entered by the patients were normalized to a 0–100 scale according to previously described protocols (8). For functional scales, higher results corresponded to a better outcome, and for symptomatic scales, higher results corresponded to more severe symptoms. A comparison of baseline scores was performed to detect differences in characteristics of normofractionated (nPBT) and hypofractionated PBT (hPBT) patients and to mitigate possible confounding effects; comparisons at the end of therapy and 3-month follow-up were made between differences in scores only.

### Statistical methods

All statistics were calculated using Stata IC 15 (StatSoft, Tulsa, OK, USA). The differences in toxicity profile between normo- and hypofractionated PBT were assessed using Pearson’s chi^2^ test for each location and toxicity group. The same method was used to evaluate possible differences in clinical variables between patients treated with both modalities. Wilcoxon’s rank-sum test was used to assess the differences in continuous variables. Additionally, logistic regression (with receiver operating characteristic (ROC) analysis for continuous variables to define the cutoff threshold) was used to identify potential risk factors for grade ≥ 3 events and toxicities without full resolution or improvement until a 3-month follow-up. Results with p-values below 0.05 were considered statistically significant. For QoL assessment, results were considered significant when p-values were below 0.05 and median score differences exceeded 10 points following the published validation studies (9, 10).

## Results

### Overall treatment tolerance

Overall tolerance of the treatment for the entire patient cohort was good. No grade 4 and 5 acute toxicities were observed. For each adverse event in an individual patient, the highest grades of 0, 1, 2, and 3 were detected in 7 (5%), 40 (28.8%), 78 (56.1%), and 15 (10.8%) patients, respectively. Of note, the overall toxicity profile was similar between primary treatment and re-irradiation (χ^2^ = 3.43, p = 0.33). The highest toxicity grades of G0, G1, G2, and G3 observed in these groups were 3 (3.4%), 22 (24.7%), 53 (59.6%), and 11 (12.4%) for primary PBT and 4 (8%), 17 (34%), 25 (50%), and 4 (8%) for re-PBT. Improvement and complete resolution of all acute toxicities between the first onset and at 3-month follow-up were seen in 105 (75.5%) and 72 (51.2%) patients, respectively. Twenty patients (14.4%) developed new-onset toxicities after therapy completion. The majority of persistent toxicities at 3-month follow-up were grades 1 and 2 (95.5%).

### Comparison of toxicity profiles

No statistically significant differences were observed between nPBT and hPBT in the majority of comparisons (14/21, 66.7%) according to treated site and affected organs. The results are displayed in **Table 3**. For H&N tumors, the only difference observed was a non-unanimous variability in skin toxicity profile—on the one hand, there were considerably more hPBT patients without any skin toxicity (27.3% *vs* 3.2%) and fewer with G1–G2 events (63.7% *vs* 95.2%) as compared to nPBT. However, a slightly greater proportion of patients had G3 reactions (9% *vs* 1.6%). Most differences (in four of seven analyses performed, 57.1%) in toxicity profile were seen in A&P and ST tumors. Hypofractionated PBT had unanimously milder skin and overall toxicity as compared to nPBT: more patients had G0–G1 events, and fewer experienced G2–G3. Furthermore, more patients treated with hPBT compared to nPBT had all observed toxicities improved (95.7% *vs* 70%) and completely resolved (87% *vs* 50%) at the 3-month follow-up. For ST tumors, the results were mixed—on the one hand, similar to the A&P tumors, less skin toxicity was seen in hPBT patients. On the other hand, all events were fully resolved until a 3-month follow-up in all patients treated with nPBT as compared to 50% of patients treated with hPBT. For all not fully resolved toxicities in this patient group, an improvement was observed.

**Table 3:** Distribution of treatment-related toxicity types according to different anatomic sites.

Toxicity	Treatment site and type of proton therapyFrequency (%)								
	Head and neck				Abdomen and pelvis			Other soft tissue	
	nPBTn = 63			hPBTn = 22	nPBTn = 20	hPBTn = 23		nPBTn = 7	hPBTn = 4
Mucosal									
G0	9.5%			9.1%	50.0%	52.2%		100.0%	75.0%
G1	22.2%			36.4%	30.0%	34.8%		0.0%	0.0%
G2	55.6%			54.5%	20.0%	13.0%		0.0%	25.0%
G3	12.7%			0.0%	0.0%	0.0%		0.0%	0.0%
Difference	χ^2^ = 4.05, p = 0.255				χ^2^ = 0.4, p = 0.817			χ^2^ = 1.93, p = 0.165	
Skin									
G0	3.2%			27.3%	30.0%	73.9%		0.0%	0.0%
G1	49.2%			36.4%	50.0%	26.1%		0.0%	75.0%
G2	46.0%			27.3%	15.0%	0.0%		71.4%	25.0%
G3	1.6%			9.1%	5.0%	0.0%		28.6%	0.0%
Difference	**χ^2^ = 14.64, p = 0.002**				**χ^2^ = 10.1, p = 0.018**			**χ^2^ = 7.4, p = 0.025**	
Other									
G0	17.5%			27.3%	35.0%	69.6%		71.4%	50.0%
G1	54.0%			27.3%	40.0%	27.3%		28.6%	25.0%
G2	27.0%			40.9%	20.0%	4.3%		0.0%	25.0%
G3	1.6%			4.5%	5.0%	0.0%		0.0%	0.0%
Difference	χ^2^ = 4.89, p = 0.18				χ^2^ = 6.43, p = 0.092			χ^2^ = 1.95, p = 0.378	
Highest toxicity grade observed									
G0	0.0%			0.0%	5.0%	26.1%		0.0%	0.0%
G1	17.5%			22.7%	40.0%	56.5%		0.0%	50%
G2	69.8%			63.6%	45.0%	17.4%		71.4%	50%
G3	12.7%			13.6%	10.0%	0.0%		28.6%	0.0%
Difference	χ^2^ = 0.34, p = 0.84				**χ^2^ = 8.51, p = 0.04**			χ^2^ = 4.83 p = 0.09	
Delayed toxicity									
Yes	19.0%	18.2%			15.0%		0.0%	0.0%	25.0%
No	81.0%	81.8%			85.0%		100.0%	100.0%	75.0%
Difference	χ^2^ = 0.01, p = 0.929				χ^2^ = 3.71, p = 0.054			χ^2^ = 1.93, p = 0.165	
Improvement within 3-month follow-up									
Yes	65.1%		77.3%		70.0%		95.7%	100.0%	100.0%
No	34.9%		22.7%		30.0%		4.3%	0.0%	0.0%
Difference	χ^2^ = 1.11, p = 0.29				**χ^2^ = 5.17, p = 0.023**				
Full resolution within 3-month FU									
Yes	38.1%			40.9%	50.0%		87.0%	100.0%	50.0%
No	61.9%			59.1%	50.0%		13.6%	0.0%	50.0%
Difference	χ^2^ = 0.05, p = 0.816				**χ^2^ = 6.93, p = 0.008**			**χ^2^ = 4.27, p = 0.039**	

nPBT, normofractionated proton beam therapy; hPBT, hypofractionated proton beam therapy; FU, follow-up. The statistically significant differences between nPBT and hPBT are highlighted in bold.

### Quality of life

In 116 of 139 (83.5%) patients, the QoL questionnaires (EORTC QLQ-C30) were completed at all time points to allow for assessment. The QoL trends in both patient groups were remarkably comparable: in 131 of 135 (97.0%) individual QoL assessment comparisons, there were no significant differences between nPBT and hPBT. Significant better role functioning between treatment start and 3-month follow-up (median = 0 for nPBT vs +16.67 for hPBT, Z = −2.94, p = 0.0033) was found in A&P hPBT patients compared to nPBT; all other scores showed no significant difference in this patient subgroup.

**Figure 1:**
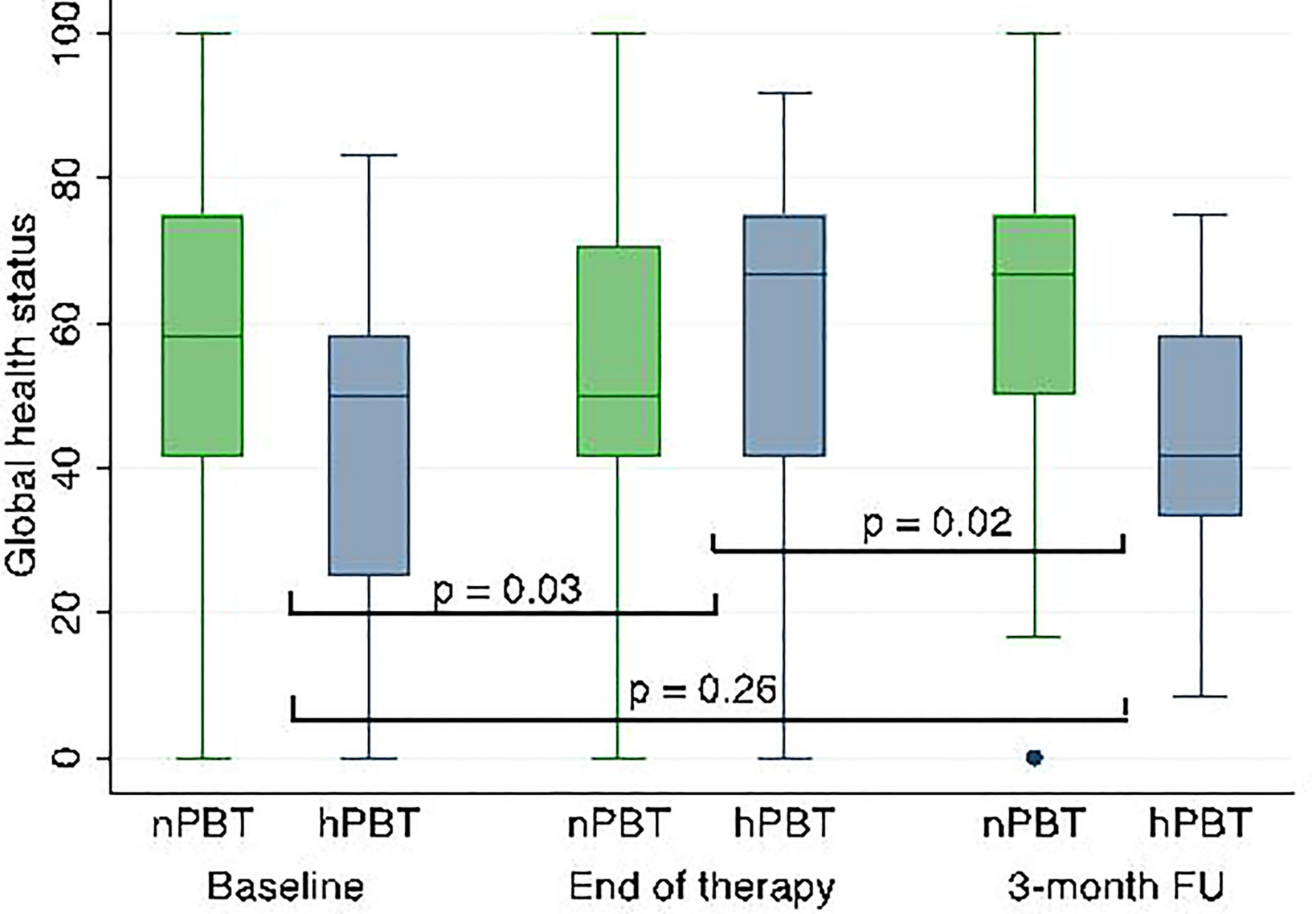
Global health status scores over time and according to fractionation groups in H&N patients; p-values refer to differences between nPBT and hPBT at corresponding time points. H&N, head and neck; nPBT, normofractionated proton beam therapy; hPBT, hypofractionated proton beam therapy.

For the baseline EORTC C30 scale in H&N patients, significant differences were observed for physical functioning (Z = 2.04, p = 0.042) and dyspnea (Z = −3.11, p = 0.0019), in both cases indicating more pre-existing symptoms and worse functioning in hPBT. No significant difference was seen in score change between hPBT and nPBT between treatment start and 3-month follow-up using the EORTC C30 scale. However, there was a significant worsening in global health status between baseline and end of treatment within the hPBT group and a significant worsening between the end of treatment and 3-month follow-up within the nPBT group. Comparing the baseline and follow-up visits for each fractionation group separately, there was no significant difference in global health status (**Figure 1**) or other domains. A difference was seen in the loss of appetite in H&N patients analyzed between start and end of therapy (median = +33.3 for nPBT and 0 for hPBT, Z = 2.32, p = 0.02), but not between treatment start and 3-month follow-up.

In the other soft tissue patient group, no significant differences in QoL values were found.

For 60 of 85 patients (70.6%) who received PBT for H&N tumors, dedicated EORTC HN35 questionnaires were additionally completed. Analogous to the results of C30 module, the H&N-specific data indicated more pre-existing symptoms in patients receiving hPBT than nPBT. These were found for seven scales: pain (Z = −2.85, p = 0.004), swallowing (Z = −2.78, p = 0.005), social eating (Z = −2.34, p = 0.019), mouth opening (Z = −2.11, p = 0.03), dry mouth (Z = −2.93, p = 0.003), sticky saliva (Z = −2.62, p = 0.009), and coughing (Z = −2.35, p = 0.018). However, these pre-existing toxicities did not translate to differences during or after therapy—in 47 of 48 individual score difference assessments (97.9%), no significant differences between nPBT and hPBT were identified. The only significant difference in score change observed during or after treatment was less prominent improvement in pain between end of therapy and 3-month follow-up for hPBT compared to nPBT (median: −8.33 *vs* −25, Z = −2.4, p = 0.016).

The median QoL baseline scores for EORTC QLQ-C30 and EORTC QLQ-HN35 scales are displayed in **Supplementary Table 1**.

### *Post-hoc* differences and risk factor analysis

*Post-hoc* analysis was performed to identify clinical and treatment characteristics with significant differences between nPBT and hPBT groups. A higher proportion of patients who previously underwent radiation was seen in all locations for hPBT compared to nPBT: 86.4% *vs* 26.9%, p < 0.0001, in H&N patients; 52.2 *vs* 0%, p < 0.0001, for A&P tumors; and 50% *vs* 0%, p = 0.039, for other locations. Additionally, for patients treated in the H&N region, the low-dose planning target volume (PTV) was significantly larger for patients treated with nPBT schemes (267.2 vs 127.82 cm^3^, p = 0.0016), and more patients were treated using simultaneous integrated boost (SIB) technique with hypofractionation (94.7% vs 27.1%, p < 0.0001). For patients with abdominal and pelvic irradiations, not only the prescription dose as shown in **Table 2** but also the respective EQD2 equivalent dose was significantly lower for hPBT than nPBT (median 67.7 *vs* 75 Gy RBE, p = 0.03), which was also expected due to low α β = 2 considered in dose prescription for prostate cancer, comprising the majority of hypofractionated patients (52.2% of hPBT vs 35% of nPBT). Also, for this group, more patients were treated with the SIB technique (71.4% *vs* 33.3%, p < 0.0001).

The clinical and treatment characteristics listed in **Tables 1** and **2** were included in risk factor analysis to identify factors potentially contributing to clinically significant conditions: grade 3 events, toxicities of delayed onset, and toxicities not fully resolved and of constant grade at 3-month follow-up. Interestingly, the factors associated with higher prescription doses (total dose, presence of macroscopic tumor, and previous irradiation) have not been associated with an increase in toxicity. For tumors of the head and neck as well as other soft tissue locations, no factors predicting the aforementioned events could be identified. Toxicities not fully resolved at 3-month follow-up were more likely to occur in elderly patients, with 69 years of age identified as the most significant cutoff age (risk 40% above *vs* 21.7% below, area under the curve (AUC) = 0.71, p = 0.042).

## Discussion

Radiotherapy regimens that reduce the number of fractions applied in selected patients are likely to become an important topic in clinical research of particle therapy. By decreasing overall beam occupation time per patient (median treatment time in our cohort: 4 vs 7.2 weeks, p < 0.0001), the hypofractionated regimen permits increasing availability for more patients given the limited and fixed overall capacity of proton centers. Due to the high cost of particle therapy centers and, consequently, their low number even in well-developed countries, this perspective seems particularly attractive for the patients in need of PBT in selected indications. However, at present, only a few studies have addressed the outcomes of schemes using once-daily fraction doses of >2.5 Gy RBE. The majority of existing clinical data on PBT hypofractionation are based on lung, liver, and prostate cancer irradiation. PBT in those locations follows the principles of photon-based stereotactic body radiotherapy (SBRT): small-to-moderate gross tumor volume size, small size or no clinical target volume (CTV) added to the gross tumor volume (GTV), and in most cases a possibility to use state-of-art image-guided radiation therapy (IGRT) and gating techniques, which further reduce the necessary ITV and PTV. The toxicity outcomes reported by the groups of Ono, Yan, and Nakamura, who used proton fraction doses between 3.2 and 4 Gy RBE for centrally located lung cancer, reported safe outcomes. Grade 3 toxicities did not exceed 4% (11–13), comparable to studies using conventional fractionation (14). Similar observations were made regarding the treatment of hepatocellular carcinoma, for which hypofractionation is the predominantly used PBT regimen: several published patient cohorts reported low and comparable toxicities for both normo- and hypofractionated groups, despite the inclusion of tumors in close proximity to critical organs for hypofractionated schemes (15). Moderate hypofractionation is, in general, used for the treatment of prostate cancer. Several studies have been published including a comparison between normo- and hypofractionated PBT: Vargas et al. compared toxicity and QoL outcomes between normo- and oligofractionated PBT (79.2 Gy RBE/44 Fx vs 38 Gy RBE/5 Fx) for low-risk prostate cancer. No difference in toxicity outcomes was observed with excellent QoL scores for the entire patient cohort (16). Other results of moderate hypofractionated PBT for prostate cancer and a single study on high-dose (75 Gy RBE in 25 fractions) definitive PBT for pelvic recurrences of colorectal adenocarcinoma reported very low rates of grade 2+ toxicities not exceeding 15%, comparable to the outcomes for abdominal and pelvic tumors of our series (17–19). The significantly better toxicity profile of hPBT compared to nPBT in our study can be possibly attributed to the lower EQD2 prescription dose used for most hPBT cases: for low-risk prostate cancer, which comprised the majority of patients in the group of abdominal and pelvic tumors, the assumed tumor α/β ratio was lower than one used to recalculate constraints for critical organs. The schemes employing similar doses have been found non-inferiorly effective and safe as compared to conventionally fractionated radiation in several clinical trials—they are in routine clinical use for patients with low- and intermediate-risk prostate cancer (20–22). A better score of hPBT compared to nPBT in role functioning in our series might be due to shortened overall treatment time since this scale focuses on work, hobbies, and leisure, and there were no differences between nPBT and hPBT patients at baseline.

For the head and neck tumor patient population in the present study, this is the first report on the comparison of early patient outcomes between normo- and hypofractionated PBT. The results of available studies for photon therapy in locally advanced head and neck tumors are mixed and not always encouraging. Bala Sankar et al. conducted a comparative study of 2D photon radiotherapy with concurrent cetuximab (66 Gy/33 Fx vs 55 Gy/20 Fx). They identified non-significant trends of more tumors showing clinical response in the hypofractionated arm (85.7% vs 57.1%) but at a cost of higher incidence of G3+G4 oral mucositis (85.7% vs 66.7%) and more frequent need of a feeding tube (71.4% vs 46.7%) (23). In a small study by De Felice et al. involving patients treated with 60 Gy in 20 fractions with concurrent cetuximab, the frequency of serious adverse events was 50%, which the authors considered unacceptable (24). An analysis of Vreugdenhil et al., which compared normofractionated and hypofractionated radiation, found no differences in outcomes, toxicities, and QoL (25). It is notable that no concurrent systemic treatment was used in our patients receiving hypofractionated PBT, and the treatment volumes were smaller as compared to nPBT. This was attributable to patient selection for hypofractionated schemes. The lack of increase in acute toxicity and QoL deterioration in hPBT patients compared to nPBT, despite a substantially higher proportion of previously irradiated patients and more severe pre-existing symptoms in this group, confirms our opinion of the early safety of moderately hypofractionated PBT. The postulated end-of-range RBE uncertainty (26) does not negatively affect acute toxicity outcomes by the use of correct planning techniques to mitigate potential consequences.

The present study has several limitations: for a full safety and efficacy profile of hypofractionated PBT, a longer follow-up is required to assess both local control, late toxicity, and QoL outcomes. In addition, the diversity of diseases treated in our cohort can be a source of potential confounding factors (previous irradiation, various volumes of healthy tissues as part of CTV, number of surgeries, and prior systemic therapy a.o.). This could possibly explain the differences between nPBT and hPBT in various soft tissue tumors. Confirmation of our observations will be best obtained by conducting a long-term comparison of outcomes in better-standardized patient subgroups. Such a project is presently underway.

## Conclusion

Moderately hypofractionated proton therapy has an acceptable early safety profile. Acute toxicity and QoL outcomes are not inferior to normofractionated proton irradiation while adding the benefits of reducing the overall treatment time. This applies also to the re-irradiation therapy regimen.

## Data availability statement

The original contributions presented in the study are included in the article/**Supplementary Material**. Further inquiries can be directed to the corresponding author.

## Ethics statement

The studies involving human participants were reviewed and approved by Amt der NÖ Landesregierung Abteilung Gesundheitswesen. The patients/participants provided their written informed consent to participate in this study. Written informed consent was obtained from the individual(s) for the publication of any potentially identifiable images or data included in this article.

## Author contributions

MP, RG, BF, EH and PG contributed to conception and design of the study. RK, PF, CF, UM, CL-C and ST organized the database. LK and MP performed the statistical analysis. JG contributed to physical parts of the study. All authors contributed to manuscript revision, read, and approved the submitted version.

## Conflict of interest

The authors declare that the research was conducted in the absence of any commercial or financial relationships that could be construed as a potential conflict of interest.

## Publisher’s note

All claims expressed in this article are solely those of the authors and do not necessarily represent those of their affiliated organizations, or those of the publisher, the editors and the reviewers. Any product that may be evaluated in this article, or claim that may be made by its manufacturer, is not guaranteed or endorsed by the publisher.
