# Supplementary material for: Normofractionated and moderately hypofractionated proton therapy: comparison of acute toxicity and early quality of life outcomes
Source: Front Oncol. 2022 Aug 16;12:962697. doi: 10.3389/fonc.2022.962697 (PMC9425455; doi:10.3389/fonc.2022.962697)
Supplement: Supplementary file 1 [file Table_1.pdf]

QoL scale item	Treatment site and type of proton therapy Median scores / score change					
	Head & Neck		Abdomen & Pelvis		Other Soft Tissue	
	nPBT n = 57	hPBT n = 18	nPBT n = 19	hPBT n = 14	nPBT n = 5	hPBT n = 3
**EORTC QLQ-C30**						
Global health status*						
Baseline	58.3	50	62.5	70.8	66.7	50
EOT vs Baseline	0	+12.5	-4.2	0	-8.3	-8.3
3m FU vs EOT	+4.2	-16.7	+8.3	0	-8.3	0
3m FU vs baseline	+8.3	-8.3	+8.3	+8.3	0	-8.3
Physical functioning*						
Baseline	86.7	66.7	100	80	93.3	80
EOT vs Baseline	0	0	0	+3.3	-6.7	-6.7
3m FU vs EOT	0	0	0	0	+6.7	+6.7
3m FU vs baseline	0	0	-6.7	0	0	-6.7
Role functioning*						
Baseline	66.7	66.7	66.7	75	66.7	66.7
EOT vs Baseline	0	0	0	+16.7	0	-16.7
3m FU vs EOT	0	0	0	0	-16.7	+16.7
3m FU vs baseline	0	0	0	+16.7	-16.7	0
Emotional functioning*						
Baseline	58.3	50	58.3	70.8	66.7	75
EOT vs Baseline	0	0	+8.3	+12.5	0	0
3m FU vs EOT	+4.2	0	0	0	0	+8.3
3m FU vs baseline	+8.3	0	+16.7	+8.3	+16.7	0
Cognitive functioning*						
Baseline	83.3	83.3	83.3	83.3	83.3	100
EOT vs Baseline	0	0	0	0	+16.7	+16.7
3m FU vs EOT	0	0	0	0	+16.7	0
3m FU vs baseline	0	0	+8.3	0	0	+16.7
Social functioning*						
Baseline	66.7	50	83.3	75	66.7	66.7
EOT vs Baseline	0	0	0	0	0	+16.7
3m FU vs EOT	0	0	0	0	0	0
3m FU vs baseline	0	0	+8.3	+16.7	0	0
Fatigue**						
Baseline	44.4	44.4	33.3	33.3	33.3	33.3
EOT vs Baseline	0	+5.5	+11.1	0	+11.1	0
3m FU vs EOT	0	0	-11.1	-11.1	-22.2	-22.2
3m FU vs baseline	0	+11.1	-5.6	-11.1	-11.1	0
Nausea**						

Baseline	0	0	0	0	0	0
EOT vs Baseline	0	0	0	0	0	0
3m FU vs EOT	0	0	0	0	0	0
3m FU vs baseline	0	0	0	0	0	0
Pain**						
Baseline	33.3	66.7	33.3	25	33.3	50
EOT vs Baseline	0	0	0	0	+16.7	-16.7
3m FU vs EOT	0	0	0	-16.7	-16.7	0
3m FU vs baseline	0	0	0	0	0	-16.7
Dyspnoea**						
Baseline	0	33.3	0	0	0	33.3
EOT vs Baseline	0	0	0	0	0	0
3m FU vs EOT	0	0	0	0	0	0
3m FU vs baseline	0	0	0	0	0	+33.3
Insomnia**						
Baseline	33.3	33.3	33.3	33.3	33.3	0
EOT vs Baseline	0	0	0	0	0	0
3m FU vs EOT	0	0	0	+33.3	0	0
3m FU vs baseline	0	0	0	0	0	0
Appetite loss**						
Baseline	0	0	0	0	0	0
EOT vs Baseline	+33.3	0	0	0	0	0
3m FU vs EOT	0	0	0	0	0	0
3m FU vs baseline	0	+33.3	0	0	0	0
Constipation**						
Baseline	0	0	0	0	0	33.3
EOT vs Baseline	0	0	0	0	0	0
3m FU vs EOT	0	0	0	0	0	0
3m FU vs baseline	0	0	0	0	0	0
Diarrhea**						
Baseline	0	0	0	0	0	0
EOT vs Baseline	0	0	0	+16.7	0	0
3m FU vs EOT	0	0	0	-33.3	0	0
3m FU vs baseline	0	0	0	0	0	0
Financial problems**						
Baseline	0	0	0	0	0	33.3
EOT vs Baseline	0	0	0	0	0	0
3m FU vs EOT	0	0	0	0	0	0
3m FU vs baseline	0	0	0	0	0	0

EORTC QLQ-HN35

	Head & Neck	
	nPBT n = 44	hPBT n = 16
Pain**		
Baseline	8.3	41.6

EOT vs Baseline	+25	+16.7
3m FU vs EOT	-25	-8.3
3m FU vs baseline	0	+8.3
Swallowing**		
Baseline	0	16.7
EOT vs Baseline	+16.7	+16.7
3m FU vs EOT	-16.7	+8.3
3m FU vs baseline	0	0
Senses problems**		
Baseline	8.3	33.3
EOT vs Baseline	+16.7	+16.7
3m FU vs EOT	-16.7	0
3m FU vs baseline	0	0
Speech problems**		
Baseline	5.6	11.1
EOT vs Baseline	+5.5	0
3m FU vs EOT	0	0
3m FU vs baseline	0	0
Social eating**		
Baseline	4.1	25
EOT vs Baseline	+16.7	0
3m FU vs EOT	-8.3	0
3m FU vs baseline	0	0
Social contact**		
Baseline	3.3	6.7
EOT vs Baseline	+6.7	+10
3m FU vs EOT	0	-6.7
3m FU vs baseline	0	0
Less sexuality**		
Baseline	25	50
EOT vs Baseline	0	0
3m FU vs EOT	0	0
3m FU vs baseline	0	0
Teeth**		
Baseline	0	0
EOT vs Baseline	0	0
3m FU vs EOT	0	0
3m FU vs baseline	0	0
Opening of mouth**		
Baseline	0	66.7
EOT vs Baseline	0	0
3m FU vs EOT	0	0
3m FU vs baseline	0	0

Dry mouth**		
Baseline	16.7	33.3
EOT vs Baseline	+16.7	0
3m FU vs EOT	0	0
3m FU vs baseline	+33.3	0
Sticky saliva**		
Baseline	0	33.3
EOT vs Baseline	+33.3	+33.3
3m FU vs EOT	0	0
3m FU vs baseline	0	0
Coughing**		
Baseline	0	33.3
EOT vs Baseline	0	0
3m FU vs EOT	0	0
3m FU vs baseline	0	0
Felt ill**		
Baseline	33.3	33.3
EOT vs Baseline	0	0
3m FU vs EOT	0	0
3m FU vs baseline	0	0
Need for pain killers**		
Baseline	100	100
EOT vs Baseline	0	0
3m FU vs EOT	0	0
3m FU vs baseline	0	0
Need for nutritional support**		
Baseline	0	0
EOT vs Baseline	0	0
3m FU vs EOT	0	0
3m FU vs baseline	0	0
Feeding tube**		
Baseline	0	0
EOT vs Baseline	0	0
3m FU vs EOT	0	0
3m FU vs baseline	0	0
Weight loss**		
Baseline	0	0
EOT vs Baseline	0	0
3m FU vs EOT	0	0
3m FU vs baseline	0	0
Weight gain**		
Baseline	0	0
EOT vs Baseline	0	0
3m FU vs EOT	0	0

3m FU vs baseline	0	0

*Functional scale: Higher score indicates better patient condition

**Symptomatic scale: Higher score indicates greater magnitude of symptom

Supplementary Table 1. Median scores of EORTC QLQ-C30 and QLQ-HN35 modules according to tumor location and fractionation scheme.
